# Severe hyposmia and aberrant functional connectivity in cognitively normal Parkinson’s disease

**DOI:** 10.1371/journal.pone.0190072

**Published:** 2018-01-05

**Authors:** Noritaka Yoneyama, Hirohisa Watanabe, Kazuya Kawabata, Epifanio Bagarinao, Kazuhiro Hara, Takashi Tsuboi, Yasuhiro Tanaka, Reiko Ohdake, Kazunori Imai, Michihito Masuda, Tatsuya Hattori, Mizuki Ito, Naoki Atsuta, Tomohiko Nakamura, Masaaki Hirayama, Satoshi Maesawa, Masahisa Katsuno, Gen Sobue

**Affiliations:** 1 Department of Neurology, Nagoya University Graduate School of Medicine, Nagoya, Japan; 2 Brain and Mind Research Center, Nagoya University Graduate School of Medicine, Nagoya, Japan; 3 Honmachi Clinic, Nagoya, Japan; 4 Department of Pathophysiological Laboratory Sciences, Nagoya University Graduate School of Medicine, Nagoya, Japan; 5 Department of Neurosurgery, Nagoya University Graduate School of Medicine, Nagoya, Japan; 6 Nagoya University Graduate School of Medicine, Nagoya, Japan; University of Texas at Austin, UNITED STATES

## Abstract

**Objective:**

Severe hyposmia is a risk factor of dementia in Parkinson’s disease (PD), while the underlying functional connectivity (FC) and brain volume alterations in PD patients with severe hyposmia (PD-SH) are unclear.

**Methods:**

We examined voxel-based morphometric and resting state functional magnetic resonance imaging findings in 15 cognitively normal PD-SH, 15 cognitively normal patients with PD with no/mild hyposmia (PD-N/MH), and 15 healthy controls (HCs).

**Results:**

Decreased gray matter volume (GMV) was observed in the bilateral cuneus, right associative visual area, precuneus, and some areas in anterior temporal lobes in PD-SH group compared to HCs. Both the PD-SH and PD-N/MH groups showed increased GMV in the bilateral posterior insula and its surrounding regions. A widespread significant decrease in amygdala FC beyond the decreased GMV areas and olfactory cortices were found in the PD-SH group compared with the HCs. Above all, decreased amygdala FC with the inferior parietal lobule, lingual gyrus, and fusiform gyrus was significantly correlated with both reduction of Addenbrooke’s Cognitive Examination-Revised scores and severity of hyposmia in all participants. Canonical resting state networks exhibited decreased FC in the precuneus and left executive control networks but increased FC in the primary and high visual networks of patients with PD compared with HCs. Canonical network FC to other brain regions was enhanced in the executive control, salience, primary visual, and visuospatial networks of the PD-SH.

**Conclusion:**

PD-SH showed extensive decreased amygdala FC. Particularly, decreased FC between the amygdala and inferior parietal lobule, lingual gyrus, and fusiform gyrus were associated with the severity of hyposmia and cognitive performance. In contrast, relatively preserved canonical networks in combination with increased FC to brain regions outside of canonical networks may be related to compensatory mechanisms, and preservation of brain function.

## Introduction

Hyposmia is a common non-motor symptom in patients with Parkinson’s disease (PD) and is frequently observed during the prodromal stage [1,2,3]. Pathologically, Lewy bodies are observed in the anterior olfactory nucleus, piriform cortex, entorhinal cortex, and particularly the amygdala, regions that are responsible for odor perception, in PD or incidental Lewy body disease [2,4]. Particularly, the amygdala is a principal olfaction hub where α-synuclein pathology may also develop even in the prodromal period^2,4^. Hyposmia is also a likely marker of future cognitive decline in PD patients [5–8]. Two prospective studies demonstrated that severe hyposmia (SH) was an independent risk factor for developing dementia in PD [6,7]. Results from positron emission tomography (PET) using ^18^F-fluorodeoxyglucose showed regional cortical dysfunction, including in the amygdala and piriform cortex, in combination with atrophy of the limbic systems in PD-SH [9]. Furthermore, acetylcholine PET demonstrated the reduced acetylcholine activity in the amygdala and the hippocampus that was correlated with the severity of hyposmia [10]. Involvement of the amygdala, particularly the basolateral nucleus, was also associated with hallucinations and dementia in PD [11–13].

However, it remains unclear how functional connectivities (FC) are involved and why cognition is preserved for several years in patients with PD-SH despite wide-ranging dysfunctional brain metabolism and brain atrophy [9]. Some compensatory mechanisms of connectivity may be responsible but this has yet to be elucidated. Recent MRI studies demonstrated clear verification of compensatory changes particularly in the early stages of neurodegenerative disease including PD [14]. Specifically, increased connectivity between the salience network and default mode network (DMN) has been shown to correlate with the improvement of working memory performance in preclinical heterozygous PD carriers [15]. Increased connectivity between the cortical areas and the inferior parietal lobule correlated with reduced behavioural impairment in tremor-dominant PD patients [16]. Furthermore, it is still not well understood why SH is a strong risk factor for developing dementia in PD.

Resting state functional magnetic resonance imaging (rsfMRI) measures spontaneous activity of the brain when an individual is not performing an explicit task. rsfMRI has been used to reveal functional network alterations in many neurological disorders, including PD. Therefore, rsfMRI could provide important information regarding FC changes in patients with PD-SH that could reveal the mechanisms underlying hyposmia and the maintenance of cognitively normal brain function in PD [17,18].

Task oriented fMRI using olfactory stimuli showed reduced neuronal activity in the amygdala and hippocampus in hyposmic PD patients [19,20]. More recently, analysis of regional homogeneity, a voxel-based measure of brain activity evaluating the similarity or synchronization between the time series of a given voxel and its nearest neighbors showed that PD with hyposmia is associated with altered functional activity not only in the traditional olfactory center, but also in some non-traditional olfactory regions of the limbic/paralimbic cortices including amygdala [21]. However, changes in both amygdala network and canonical resting state networks (RSNs) in PD-SH have not been assessed.

Here, we investigated brain atrophic changes and FC alterations in patients with PD-SH, patients with no/mild hyposmia (PD-N/MH), and healthy controls (HCs) using voxel-based morphometry (VBM) and rsfMRI, respectively. We aimed to elucidate the pathophysiology of the link between SH and brain network changes using seed-based connectivity (SC) analysis with the amygdala as the seed region of interest (ROI). We also used independent component analysis (ICA) and dual regression analysis to assess changes in the canonical RSNs associated with cognitive function.

## Materials and methods

### Participants

We investigated patients with PD, diagnosed according to the UK Brain Bank criteria, who were referred to the Department of Neurology at Nagoya University. The patients were in Hoehn and Yahr (HY) Stages I–III, were 55–75 years old at examination, and experienced disease onset after the age of 40. We excluded patients with any history of other neurological or psychiatric diseases, focal deep white matter abnormalities characterized by hyperintensities in T2-weighted MRI images that were more severe than Grade 2 based on the Fazekas hyperintensity rating system [22], or any family history of parkinsonism. Patients with tremor-dominant PD were excluded to minimalize motion artifact. Written informed consent was obtained from all participants. The study was approved by the Ethical Committee of Nagoya University Graduate School of Medicine.

All patients were assessed using the Odor Stick Identification Test for the Japanese (OSIT-J; Daiichi Yakuhin, Co., Ltd., Tokyo, Japan) [23] and Addenbrooke’s Cognitive Examination-Revised (ACE-R) [24] for their odor-identification performance and general cognitive function. The OSIT-J consists of 12 odorants familiar to the Japanese and is widely used for evaluating olfactory function in PD patients. The OSIT-J score in HCs was 8.3 ± 2.2 [25]. We classified PD patients having OSIT-J scores < 4 (lower than the mean OSIT-J score observed in HCs minus 2 standard deviations [SDs]) as PD-SH; PD patients with OSIT-J scores ≥ 6 (greater than the mean score minus 1 SD) were classified as PD-N/MH. The ACE-R is a battery that evaluates six cognitive domains (orientation, attention, memory, verbal fluency, language, and visuospatial ability) and provides discriminative power for the diagnosis of dementia in patients with PD [26].

We excluded all patients who had an ACE-R score ≤ 88, indicating mild cognitive impairment, and those who had hallucinations, psychotic behavior, dopamine dysregulation syndrome, depressed mood, anxious feelings, or apathy based on complete evaluations of the Japanese version of the Movement Disorder Society (MDS)-sponsored revision of the Unified Parkinson's Disease Rating Scale (MDS-UPDRS) [27]. MDS-UPDRS Part III was evaluated at “on” time.

Eighteen patients with PD-SH underwent further MRI evaluation. Three patients who moved more than 3 mm during scanning were excluded from the statistical analysis; the final analysis included 15 patients with PD-SH. We also enrolled 15 patients with PD-N/MH, and 15 HCs without neurological disease, family history of PD, or hyposmia, and with an ACE-R score > 88 in the study (Table 1).

**Table 1:** Patient demographics.

	PD with severe hyposmia	PD with no/mild hyposmia	Healthy controls	P values
Number	15	15	15	NS
Male/Female	7/8	6/9	7/8	NS
Age at examination	70.7(4.8)	64.4 (7.2)	63.3(5.2)	p = 0.01
Duration (y)	5.9(3.7)	6.1(3.2)	NA	NS
ACE-R	94.3(3.4)	96.1(3.1)	97.3(2.9)	NS
MMSE	29.1 (1.1)	29.0 (1.3)	29.5 (0.6)	NS
OSIT-J	1.7(1.1)	7.5(1.5)	10.4 (1.3)	p < 0.0001
Laterality (R/L/B)	7/7/1	4/11/0	NA	NS
LEDD	455.9(377.8)	394.7(277.0)	NA	NS
Hoehn and Yahr stages	2.0(0.4)	2.0(0.5)	NA	NS
MDSUPDRS-I	5.1(4.2)	4.6(3.9)	NA	NS
MDSUPDRS-II	7.5(4.5)	10.3(6.5)	NA	NS
MDSUPDRS-III	19.3(7.9)	21.2(9.4)	NA	NS
MDSUPDRS-IV	1.9(3.5)	2.0(3.3)	NA	NS

Data are means ± standard deviation (SD). PD, Parkinson’s disease; ACE-R, Addenbrooke’s Cognitive Examination-Revised; MMSE, mini-mental state examination; R, right side predominant; L, left side predominant; B, bilateral; LEDD, levodopa equivalent dose; MDS-UPDRS, Movement Disorder Society-Sponsored Revision of the Unified Parkinson’s Disease Rating Scale; NS, not significantly different; NA, not applicable.

Comparisons of the differences in gender and laterality were performed using the chi-square test. Hoehn and Yahr stages and the MDSUPDRS-IV scores were compared using the Student’s t-test. To compare duration, MDSUPDRS-I/II/III scores and LEDD, we used the Mann- Whitney U test. To compare age at examination, MMSE and ACE-R, we used one-way analysis of variance. To evaluate differences in OSIT-J we used in the Kruskal-Wallis test.

### MRI

All MRI scans were performed using a Siemens Magnetom Verio (Siemens, Erlangen, Germany) 3.0 T scanner with a 32-channel head coil at Nagoya University’s Brain and Mind Research Center. High resolution T1-weighted images (repetition time [TR] = 2.5 s, echo time [TE] = 2.48 ms, 192 sagittal slices with 1-mm thickness, field of view [FOV] = 256 mm, 256 × 256 matrix size) were acquired for anatomical reference. Total scanning time for the T1-weighted images was 349 seconds. rsfMRI scans (8 min, eyes closed) were also acquired (TR = 2.5 s, TE = 30 ms, 39 transverse slices with a 0.5-mm inter-slice interval and 3-mm thickness, FOV = 192 mm, 64 × 64 matrix dimension, flip angle = 80 degrees). MRI scanning was performed while the PD patients were in the “ON” medication state.

### Age and gender correction

To correct for age and gender, we followed the method proposed by Dukart et al. [28]. Using only data from the HCs, a regression model for age and gender was generated at each voxel. Using the estimated regression coefficients, age and gender were then regressed out from the data of both the HC and patient groups. This method was applied in all subsequent analyses.

### VBM

Three-dimensional T1-weighted images (T1WI) were analyzed using SPM8 (Wellcome Trust Center for Neuroimaging, London, UK) running on MATLAB (MathWorks, MA, USA). The T1WI were first segmented into component images, normalized to the Montreal Neurological Institute (MNI) space using diffeomorphic anatomical registration using exponentiated Lie algebra (DARTEL) [29], modulated to preserve the total amount of signal from each region, resampled to a voxel resolution of 2 × 2 × 2 mm^3^ and spatially smoothed using an 8-mm full width at half maximum (FWHM) isotropic Gaussian filter. Group comparisons were performed on the preprocessed gray matter images with the total intracranial volume included as a covariate of no interest.

### Amygdala-based FC analysis

We employed SC analysis of the amygdala to examine the underlying network changes in PD-SH using the Juelich Histological Atlas, which provides a precise parcellation and structurally homogenous ROIs of the amygdala based on cytoarchitectural properties of the human brain [30].

To investigate changes in FC of the amygdala to the rest of the brain, we performed SC analysis using rsfMRI datasets. SPM8 was used to preprocess the functional images. For each participant's data, the first 5 volumes were discarded to account for the initial image inhomogeneity. The remaining images were then realigned to the mean functional image, co-registered to the participant's structural image, normalized to the MNI stereotactic template, resampled to a 2 × 2 × 2-mm^3^ voxel resolution, and smoothed using an 8-mm FWHM Gaussian filter. We further regressed out the six estimated motion parameters, the mean signals from selected regions of interest within cerebrospinal fluid and white matter, and time-shifted versions (t + 1, t − 1) of these signals to remove the effects of head motion and other physiological noise. Finally, the data were band-pass filtered (0.01–0.08 Hz) using Matlab’s *fir1()* bandpass filter function.

Using the Juelich Probabilistic Atlas, we divided the amygdala into six subregions, the left and right centromedial amygdala, left and right laterobasal amygdala, and left and right superficial amygdala, and used these as seed regions (S1 Fig). To compute the connectivity, time series within each seed region were extracted from the preprocessed resting state data and the mean was computed. The resulting mean series was then correlated with the time series from all voxels within the brain. The correlation coefficients were then converted into z-scores using the Fisher Transform. The correlation coefficients and z-scores were computed using an in-house Matlab script (source code in S6 Fig). Two-sample *t*-tests were performed to examine changes in connectivity between groups using SPM8 with the z-score images used as inputs. Specifically, we performed the following comparisons: HC vs PD-N/MH, HC vs PD-SH, and PD-N/MH vs PD-SH. The resulting statistical maps were thresholded at p < 0.05, corrected for multiple comparisons using cluster-level family-wise error (FWEc), with the cluster-defining threshold (CDT) set to p = 0.001.

Using SPM8, we also performed regression analyses to identify the relationship between the age-and-gender-corrected z-scores and OSIT-J as well as ACE-R scores for each seed ROI. We investigated the relationship of z-scores and OSIT-J scores within regions that showed significant connectivity differences between groups to show that some connectivity changes were also correlated with OSIT-J scores. We masked the result of the regression analyses using contrasts obtained from group comparisons (e.g., contrast map from HC > PD-SH). The result of the ACE-R regression analysis was also masked by the contrast maps obtained from group comparisons and OSIT-J regression analysis to identify regions that showed: 1) significant connectivity changes between groups, 2) significant connectivity correlation with the OSIT-J score, and 3) significant correlation with the ACE-R score. These regions could play an important role in the transition from normal cognition to cognitive impairment in patients with PD-SH.

### Canonical RSN analysis

Changes in well-known canonical RSNs were investigated using ICA. Functional and anatomical images were pre-processed using the FSL software package [31]. Each participant’s T1WI was skull stripped using the FSL Brain Extraction Tool [32] and then normalized to the MNI 152 template using FLIRT [26]. Functional images were realigned relative to a representative volume from the series, co-registered to the extracted brain, and spatially smoothed using an 8-mm FWHM Gaussian filter. The smoothed images were then normalized to the MNI 152 standard space using the same transformation matrix applied in normalizing the extracted brain image. The normalized images were resampled to an isotropic voxel resolution of 2 × 2 × 2 mm^3^.

To correct for physiological noise and other nuisance signals, we also regressed out the estimated motion parameters, the mean signals from selected regions of interest within the cerebrospinal fluid and white matter, and temporally shifted versions of these signals. In terms of head motion, the estimated mean relative displacement for each group was: HC = 0.11 mm (SD = 0.069 mm), PD-N/MH = 0.09 mm (SD = 0.066 mm), and PD-SH = 0.09 mm (SD = 0.048 mm). No significant difference in head motion was observed between groups.

The preprocessed resting state datasets from the 45 participants were temporally concatenated, and group ICA was performed using MELODIC, a component of the FSL package. We extracted 30 independent components and selected 10 canonical RSNs with the greatest overlap to established RSN templates, including the left and right executive control networks, the dorsal and ventral default mode networks, as well as the salience, precuneus, high visual, primary visual, and visuospatial networks, which are all associated with cognitive functions, such as working memory, salience processing, episodic memory, visuospatial attention, and vision, for further analysis[33].

Differences in these networks between the patient and HC groups were examined using dual regression analysis [34]. Statistical analyses of the different component maps were performed using nonparametric permutation testing with 5,000 permutations. A threshold-free cluster enhancement technique was used to control for multiple comparisons [35]. All reported statistical maps were corrected for multiple comparisons with FWE p < 0.05. Age and gender were regressed out from individual RSNs using the method described earlier before statistical analysis.

## Results

### Patient demographics

Gender, disease duration, ACE-R, and MMSE scores did not significantly differ among the PD-SH, PD-N/MH, and HC groups (Table 1). Age at examination was greater in the PD-SH group than in the PD-N/MH and HC groups.

### Gray matter volume (GMV) changes

#### Decreased GMV

**Fig 1:**
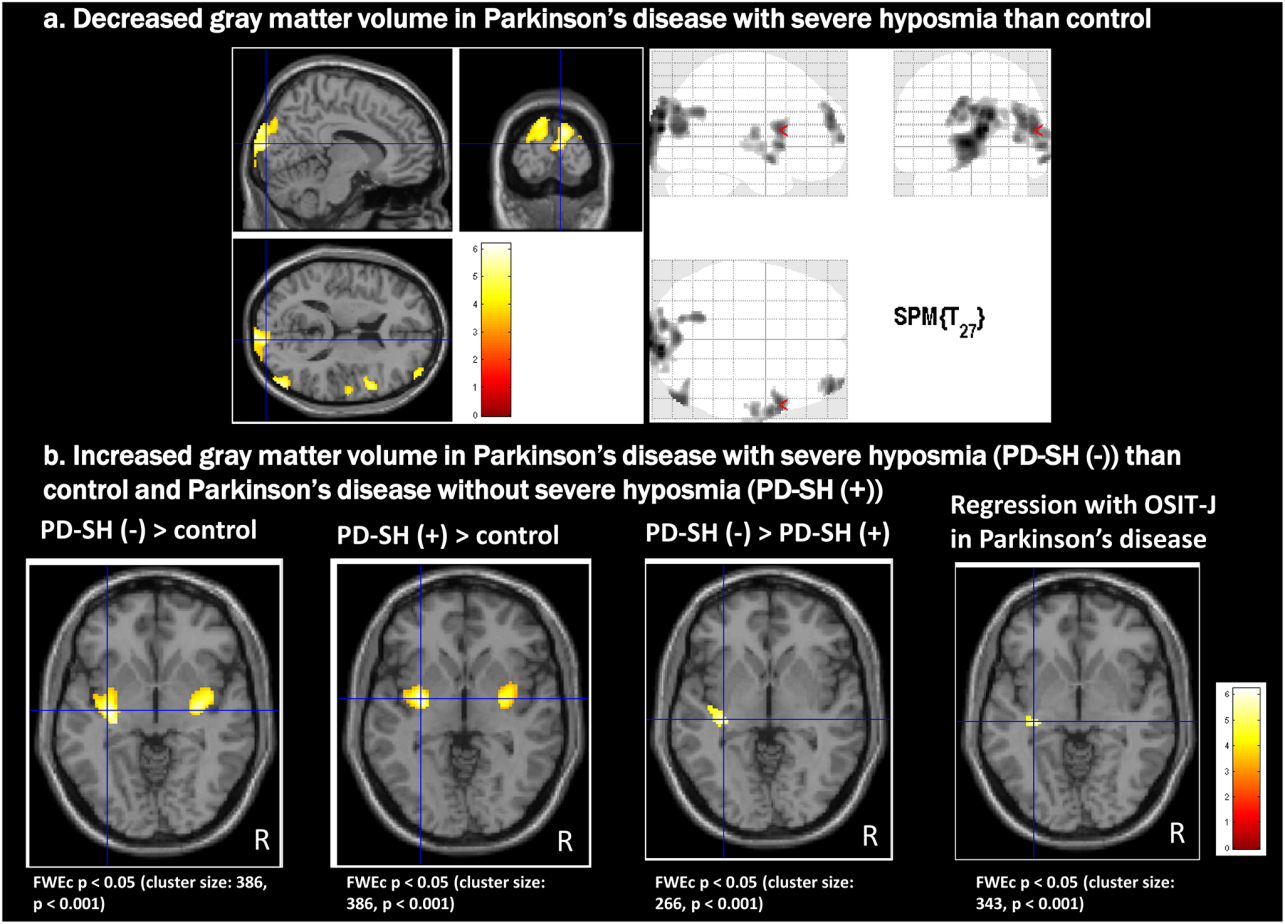
Voxel-based morphometry (VBM) findings in patients with Parkinson’s disease (PD). (A) Decreased gray matter volume (GMV) in the right cuneus, associative visual area, precuneus, middle temporal gyrus, superior frontal gyrus, middle frontal gyrus, inferior frontal gyrus corresponding to the operculum, superior temporal gyrus, precentral gyrus, and middle temporal gyrus in the PD with severe hyposmia (PD-SH) group relative to healthy controls (HCs). (B) Increased GMV in the PD with no/mild hyposmia (PD-N/MH) group relative to HCs and the PD-SH group. Both PD groups showed increased GMV in the bilateral posterior insula and its surrounding regions. The PD-SH group also showed a significant increase in GMV in a more restricted area of the posterior insula compared with those with the PD-N/MH group. The regression analysis showed significant correlations between GMVs in the same regions and scores on the Odor Stick Identification Test for the Japanese (OSIT-J) in patients with PD. Maps were thresholded at p < 0.05, corrected for multiple comparisons using cluster-level family-wised error correction (FWEc) with a cluster-defining threshold (CDT) set at p = 0.001.

Compared with the HCs, the PD-SH group showed decreased GMV in the bilateral cuneus, right associative visual area, precuneus, middle temporal gyrus, superior frontal gyrus, middle frontal gyrus, inferior frontal gyrus corresponding to the operculum, superior temporal gyrus, precentral gyrus, and middle temporal gyrus (Fig 1a). The PD-N/MH group showed no significant GMV decreases compared with either the HCs or patients with SH.

#### Increased GMV

Compared with HCs, the PD-N/MH group showed increased GMV in the bilateral posterior insula and its surrounding regions (Fig 1b). The PD-SH group also showed a significant increase in GMV in a more restricted area of the posterior insula compared with those with HC. The regression analysis showed a significant positive correlation between GMV in these regions and OSIT-J scores in all PD patients (PD-N/MH + PD-SH).

### RSNs and connectivity analysis

#### Amygdala-based FC analysis

**Fig 2:**
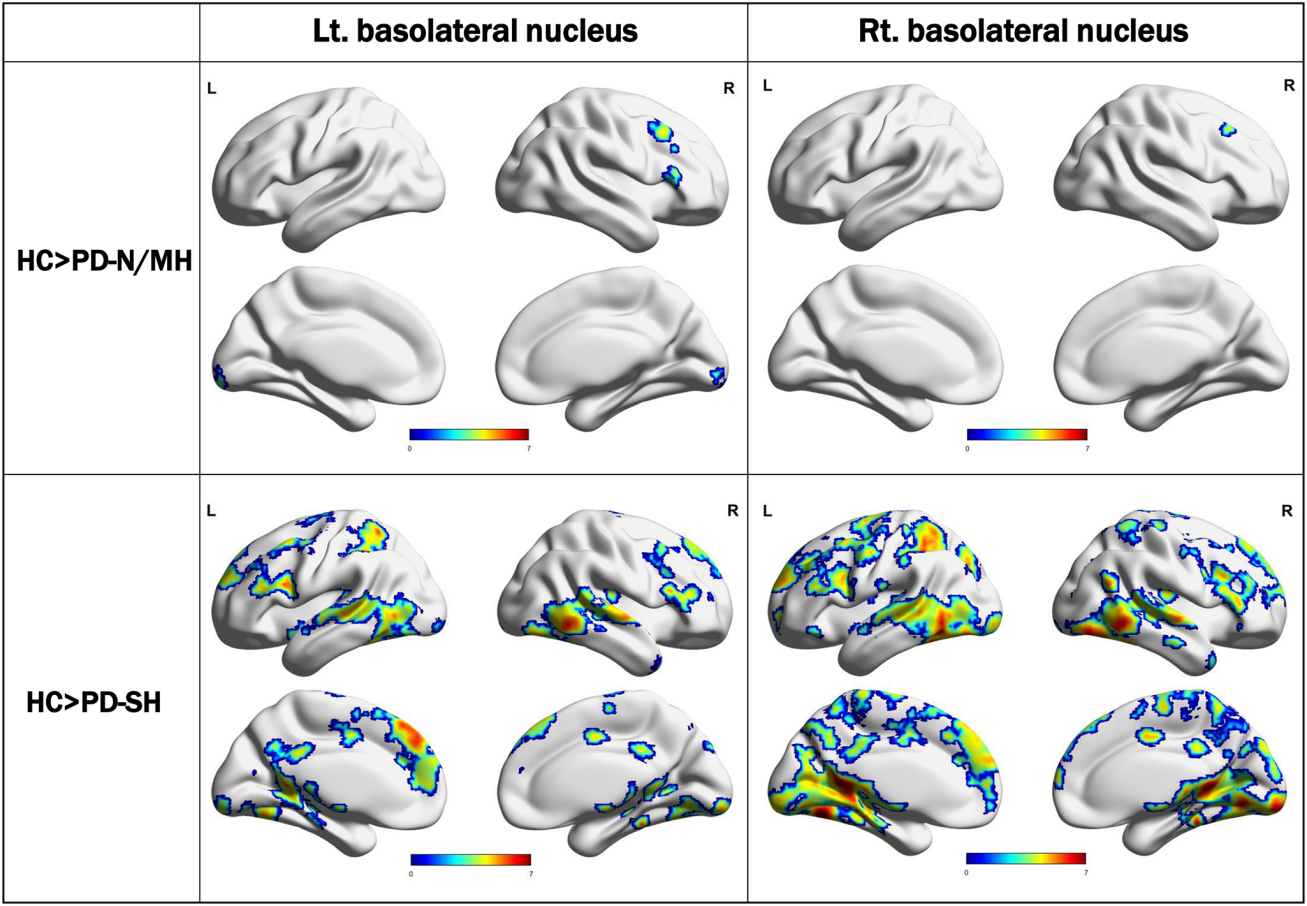
Seed-based connectivity analysis from the basolateral nucleus of amygdala and other brain voxels in the PD-N/MH and PD-SH groups. In the PD-SH group, seed-based connectivity analysis of the basolateral nucleus of the amygdala showed widespread decreases in functional connectivity (FC) to other brain regions. Areas of decreased FC were more extensive than those of decreased GMV.

In the PD-SH group, SC analysis of the basolateral nucleus of the amygdala showed widespread decreases in FC to other brain regions (Fig 2). Areas of decreased FC were more extensive than those of decreased GMV. With the superficial nucleus as the seed region, significant decreases in FC with widespread cerebral regions were observed (S2 Fig). Similarly, FC changes of the centromedial nucleus were found, but only to a relatively limited number of cerebral regions compared with the basolateral and superficial nuclei (S2 Fig). The full list of areas including MNI coordinates, z-values, and cluster size, among others, showing significant difference in connectivity for all group-level comparisons and all seed ROIs is given in S7 Fig.

The PD-SH group also showed decreased FC between the right centromedial amygdala and mainly dorsolateral prefrontal cortex and parietal cortex compared with the PD-N/MH group (S3 Fig). However, there were no significant differences in FC of other amygdala seeds of interest, suggesting that the PD-N/MH group might have mild but similar FC impairments compared with the PD-SH group.

In the PD-N/MH group, the SC analysis showed decreased FC of the three amygdala seeds of interest, although the spatial distributions of the decreased FC were significantly limited compared with those of the PD-SH group. Compared with HCs, no significant increases in FC among the three amygdala seeds of interest were observed in either the PD-SH or PD-N/MH group.

**Fig 3:**
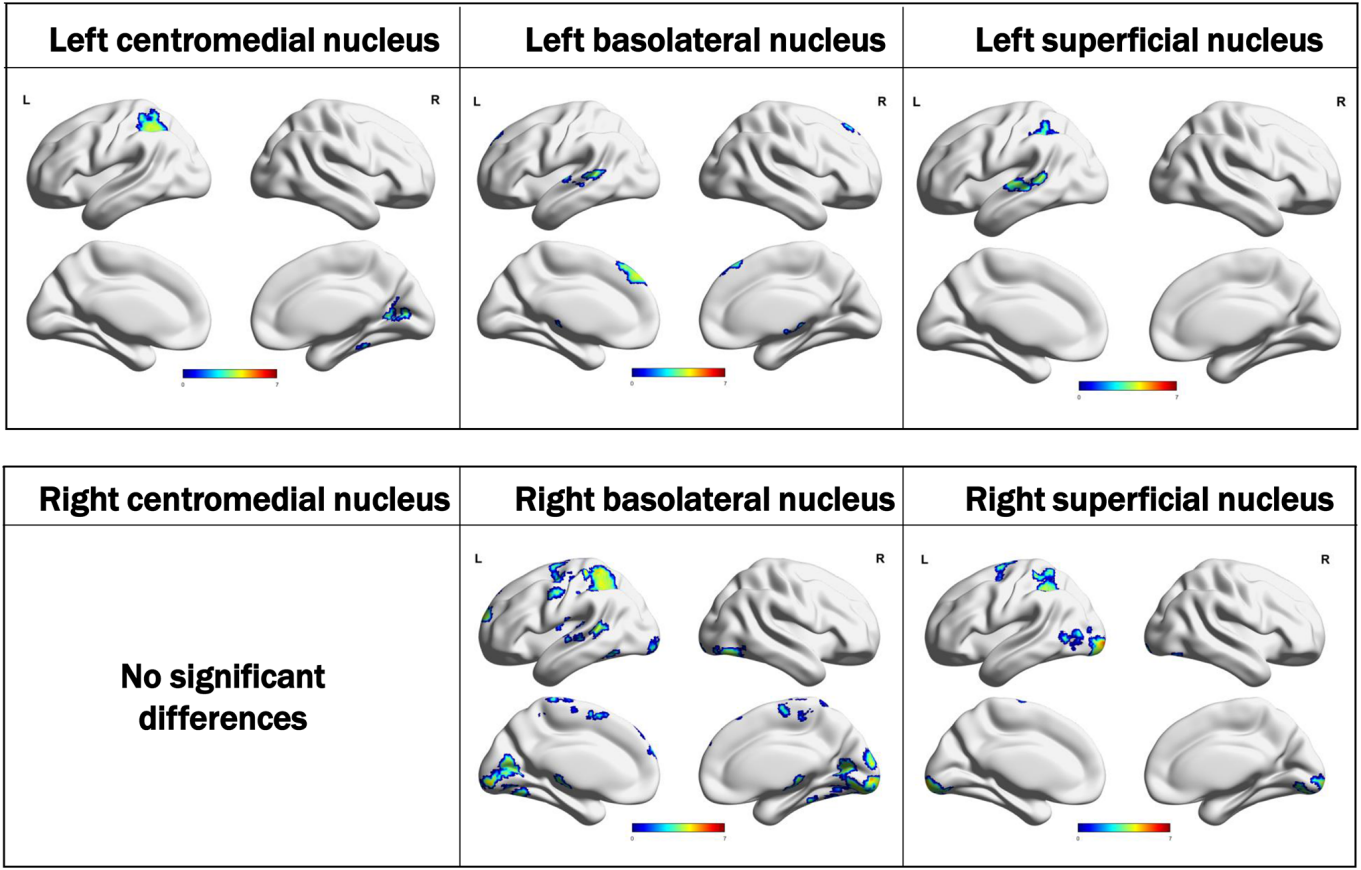
Regression analysis of amygdala connectivity with the OSIT-J and Addenbrooke’s Cognitive Examination-Revised (ACE-R) scores in all participants. The regression analysis in revealed that amygdala FC was significantly correlated with the inferior parietal lobule, lingual gyrus, fusiform gyrus, and superior and middle temporal gyrus when the OSIT-J score was masked by the contrast of the group comparison result and also when the ACE-R score was masked by the contrast of the group comparison and OSIT-J results in all participants. All maps were corrected for multiple comparisons using FWEc p < 0.05 and CDT p = 0.001.

Regression analysis masked by the contrast of the group comparison result in all participants showed several regions with FC values that were significantly related with the OSIT-J score (Fig 3a). Within the regions that not only showed significant differences between PD-SH and HC but also a significant correlation with the OSIT-J score, the inferior parietal lobule, lingual gyrus, fusiform gyrus, and superior and middle temporal gyrus showed a significant correlation with decreased ACE-R scores (Fig 3b).

#### Canonical RSN analysis

**Fig 4:**
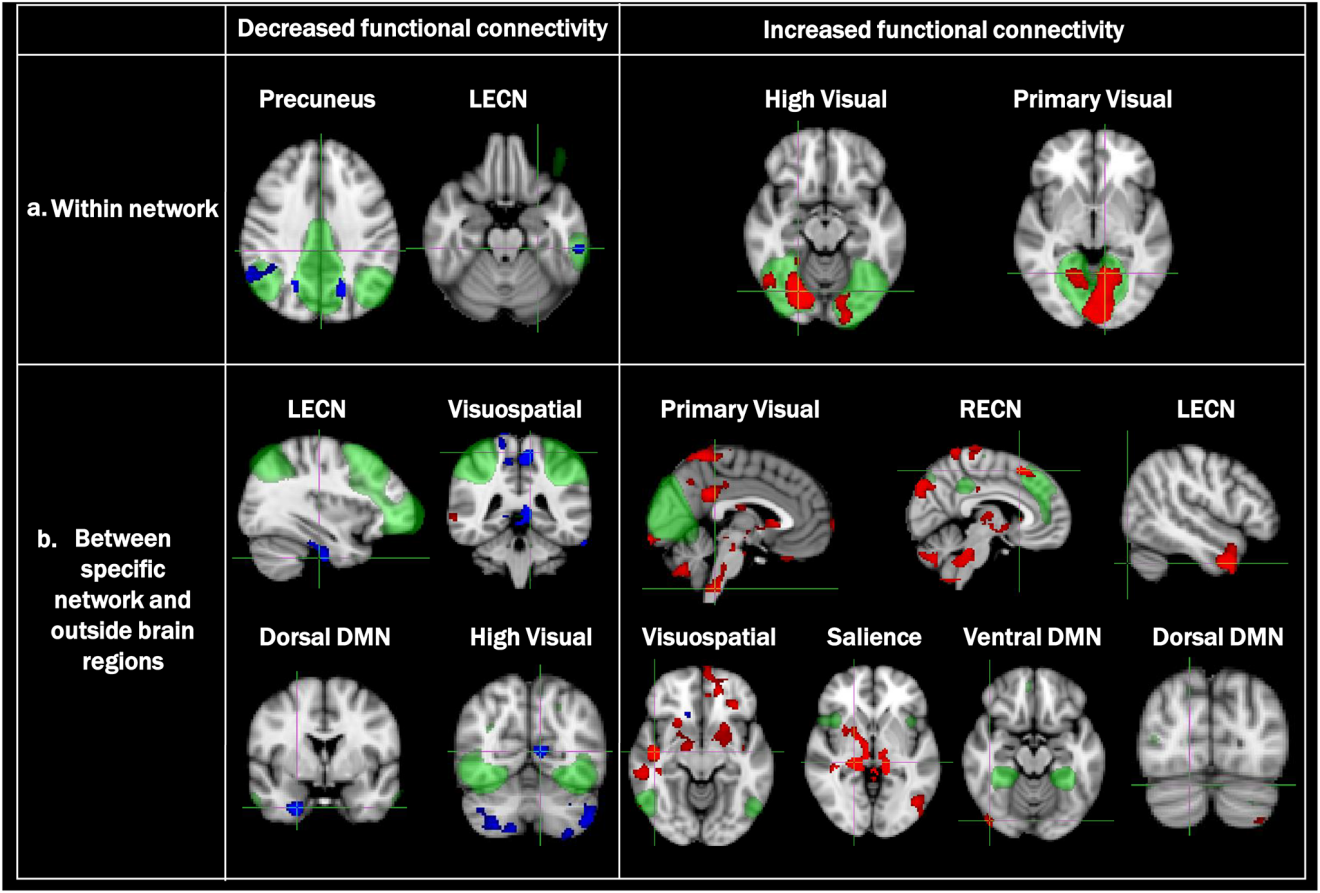
Independent component analysis (ICA) in the PD-SH group compared with HCs. (a) Analysis of FC changes, relative to controls, within canonical resting state networks (RSNs; green) showed significantly decreased connectivity within the precuneus network (blue) but increases within the primary and high visual networks (red). (b) Analyses of FC changes, relative to HCs, between canonical RSNs and brain regions outside of these networks showed decreased connectivities between several networks, including the left executive control network (ECN), dorsal default mode network (DMN), high visual, and visuospatial brain regions, and regions outside of these networks. More prominent increased connectivity changes were observed than decreased changes between canonical networks, including the primary visual network, left and right ECNs, visuospatial network, salience network, and dorsal/ventral DMNs, and brain regions outside of each network. A threshold-free cluster enhancement technique was used to control for multiple comparisons (FWE p < 0.05).

FC changes within canonical RSNs. Fig 4a shows FC changes within canonical RSNs relative to HCs. The PD-SH group showed significant decreases in FC within the precuneus network and significantly increased FC within the high and primary visual networks. There were no within-network connectivity changes in the other RSNs. No significant FC changes were observed between PD-SH and PD-N/MH.

FC changes between canonical RSNs and brain regions outside of canonical networks. Fig 4b shows FC changes, relative to HCs, of several canonical RSNs with regions outside of these networks. In the PD-SH group, several RSNs, including the precuneus, left executive control, visuospatial, dorsal default mode, and high visual networks, showed decreased FC relative to HCs. In the precuneus, the changes were relatively widespread, while the changes were more localized and limited in others (e.g., the left executive control network, visuospatial, dorsal default mode).

Conversely, significant increases relative to HCs were observed in the FC to brain regions outside of several canonical networks, including the primary visual, executive control, visuospatial, salience, and default mode networks, in patients with SH. Of these, the primary visual, right executive control, salience, and visuospatial networks showed more widespread changes. In others, the changes were localized to a few regions. The PD-N/MH group did not significantly differ relative to HC in FC within any of the networks or to brain regions outside of the canonical networks. No significant FC changes were observed between PD-SH and PD-N/MH.

## Discussion

We reported novel findings regarding relationships between hyposmia and decreased brain volume and decreased FC in PD patients. The PD-SH group had decreased GMV and FC compared to HCs: 1) atrophic changes in brain regions associated with higher olfactory function, including the operculum, temporal cortex, and the parietal cortex-precuneus; and 2) widespread FC decreases of amygdala nuclei with other brain regions, especially the inferior parietal lobule, lingual gyrus, fusiform gyrus, and superior and middle temporal gyrus. These regions showed a significant correlation with both decreased OSIT-J and ACE-R scores; 3) decreased FC within the precuneus network and among canonical RSNs and other brain regions outside of these networks. We also identified relationships between hyposmia and increased GMV and enhanced FC: 4) the PD-SH and PD-N/MH groups showed increased posterior insula volume relative to HCs; 5) the PD-SH group showed increased FC within the high and primary visual networks; and 6) more widespread increased connectivity between canonical RSNs and brain regions outside of these canonical networks. As compared to PD-N/MH group, the degree and extent of FC decreases in PD-SH group were greater but statistical differences were only observed in FC from the right centromedial amygdala, suggesting that the PD-N/MH group might have mild but similar FC impairments compared with the PD-SH group.

### Relationships of hyposmia with decreased amygdala FC

The FC among all three amygdala subregions as seed ROIs and other brain voxels showed highly impaired FC in the PD-SH group. Compared with other subregions of the amygdala, FC of the basolateral nucleus was the most widely affected in PD-SH. Regression analysis showed a significant relationship between decreased ACE-R scores in PD and reduced amygdala FC to the inferior parietal lobule, lingual gyrus, fusiform gyrus, and superior/middle temporal gyrus. The inferior parietal lobule, lingual gyrus, and fusiform gyrus would also be associated with olfactory recognition [36–38]. Interestingly, these areas and the amygdala did not show GMV reduction in either the PD-SH or PD-N/M group compared with the HCs in this study; however, significant atrophic changes are observed in PD patients with hallucination, mild cognitive impairment, and dementia [39–42]. These results suggested that decreased FC between the amygdala and inferior parietal lobule, lingual gyrus, and fusiform gyrus may be early important changes associated with future development of dementia in PD.

### Relationship of hyposmia with decreased FC in canonical networks

As compared to amygdala FC, the ICA did not show widespread decreased FC in most canonical networks; within-network FC decreases were observed in the bilateral precuneus and right inferior parietal lobule in the precuneus network and the left temporal gyrus in the left executive control network. The PD patients included in this study were cognitively normal (ACE-R scores ≥ 89) despite SH and widespread disruption of the amygdala and whole-brain FC. These relatively intact canonical networks may underlie normal cognition in patients with PD-SH. The preservation of canonical networks may play an important role in maintaining cognitive function despite disease progression.

### Relationship of hyposmia with decreased GMV

Using cerebral blood flow PET, Savic et al. identified specific areas associated with higher olfactory functions, such as the passive smelling of odors, discrimination of odor intensity, discrimination of odor quality, and odor recognition memory [36]. The bilateral precuneus/parietal cortex and right operculum, which showed decreased GMV in our PD-SH group, were associated with odor recognition memory and discrimination of odor quality. Because OSIT-J can evaluate both passive smelling of odors and discriminate odor quality and recognition memory [23], limited but decreased GMV in these regions may be associated with the underlying pathophysiology of the low OSIT-J scores in these patients.

### Relationship of hyposmia with increased GMV and increased FC

Interestingly, significant increases in GMV relative to HCs were observed mainly in the posterior insula in both PD groups. Moreover, the OSIT-J scores of all the participants were positively correlated with these increased GMVs. However, the role of an increased GMV remained unclear. This increased GMV could be due to many pathological changes including increased number of cells, larger cells, and swelling, among others.

The primary olfactory system, including the olfactory bulb, amygdala, and piriform cortex, connected with the insular cortex, is involved during the premotor stage of PD [3]. These chronic and longstanding primary olfactory system involvements may cause remodeling of neuronal circuits, resulting in morphological changes and aberrant neural plasticity in the olfactory area of the insular cortex; the anterior part of the insular cortex plays an important role in olfaction [42]. Increased hippocampal GMV prior to progressive atrophy was also reported in familial Alzheimer’s disease [43]. Although the increased GMV in the posterior insula may occur as a reciprocal change due to involvement of the anterior part, further studies are needed to clarify the pathophysiology of increased GMV in the posterior insula.

Increased FC of canonical RSNs, such as the high visual, primary visual, executive control, visuospatial, salience, and default mode networks, to brain regions outside of these canonical networks were also observed in the PD-SH group compared with the HCs. The pathophysiological background of increased FC between these canonical networks and outside brain regions was imprecise. Since both patients with PD-SH and PD-N/MH had similar cognitive performance as assessed by their ACE-R scores in this study, wide-ranging increased FC may play a role in preserving multiple brain functions in PD patients, including cognition. Subsequent failure of compensatory processes and the continued degeneration of the overall network structure might lead to development of further olfactory and eventual cognitive failure or dementia in these patients. However, increased FC may be associated with not only compensation but also dedifferentiation. Further prospective observations or task related fMRI studies are needed to elucidate whether the observed increased connectivity is related to maladaptative or adaptative neural responses to neurodegenerative changes.

### Relationship between dominant side of PD for the patients and imaging results

In this study, proportion of the PD patients with right-side predominant was higher in PD-N/MH than in PD-SH, but there were no significant statistical differences in the estimated scores using Chi-squared test. Right-side predominant abnormalities of VBM and amygdala-based FC findings observed in PD-SH as compared to HC could not be explained by the laterality assessed by motor symptoms. Although this may be due to the limitation of the patients' number, the relationship between laterality of motor symptom and cognition is still controversial [44–48]. Further studies will be needed to elucidate the relationship of laterality between motor symptom and cognition.

### Limitations

Since this is a cross-sectional study, we could not verify whether our patients with PD-SH had higher risk of developing dementia in the future as compared to those with PD-N/MH. Future longitudinal investigation will be necessary to fully address the relationship between the connectivity changes associated with SH and cognitive decline.

In conclusion, we demonstrated extensive decreased amygdala FC in PD-SH. Particularly, decreased FC between the amygdala and inferior parietal lobule, lingual gyrus, and fusiform gyrus were associated with the severity of hyposmia and cognitive performance. Contrary, increased GMV in the bilateral posterior insula and its surrounding regions and relatively preserved canonical networks in combination with increased FC to brain regions outside of canonical networks may be related to compensatory mechanisms, and maintenance of cognition. Further prospective studies need to clarify whether increased GMV and FC may be a potential mechanism to compensate for the disruption of olfactory function-related networks in PD.

## Supporting information

S1 Fig Juelich Probabilistic Atlas. The amygdala was divided into six subregions: left and right centromedial amygdala, left and right laterobasal amygdala, and left and right superficial amygdala. These were used as seed regions.
(TIF)

S2 Fig Seed-based connectivity (SC) analysis between the amygdala nuclei and other brain voxels. In the Parkinson’s disease with severe hyposmia (PD-SH) group, the amygdala nuclei showed widespread decreases in functional connectivity (FC) with other brain areas compared with healthy controls (HCs). The PD-N/MH group also showed decreased FC compared with HCs in three regions of interest (ROIs); however, the extent of these abnormal connectivity regions was limited compared with those of the PD-SH group versus HCs. All maps were corrected for multiple comparisons using FWEc p < 0.05 with CDT p = 0.001.
(TIF)

S3 Fig Comparison of seed-based connectivity (SC) analysis between the amygdala nuclei and other brain voxels between Parkinson’s disease with severe hyposmia (PD-SH) and PD with no/mild hyposmia (PD-N/MH). The PD-SH group showed decreased FC between the right centromedial and mainly dorsolateral prefrontal cortex and parietal cortex including left inferior frontal gyrus/BA9, postcentral gyrus, precentral gyrus, inferior parietal lobule/BA40, middle frontal gyrus/BA9, right inferior parietal lobule, postcentral gyrus/BA3, precentral gyrus, and paracentral lobule compared with the PD-N/MH group. However, there were no significant differences in FC of other amygdala seeds of interest.
(TIF)

S4 Fig Regression analysis of amygdala connectivity with the Odor Stick Identification Test for Japanese (OSIT-J) scores in all participants. We investigated the relationship of z-scores and OSIT-J scores within regions that a showed significant connectivity difference between groups to demosntrate that some connectivity changes were also correlated with OSIT-J scores. We masked the result of the regression analyses using contrasts obtained from group comparisons (e.g., contrast map from HC > PD-SH). Most of the connectivity changes were correlated with the OSIT-J scores, signifying that these changes were related to hyposmia in all participants. All maps were corrected for multiple comparisons using cluster-level family-wise error (FWEc) p < 0.05 and cluster-defining threshold (CDT) p = 0.001.
(TIF)

S5 Fig Regression analysis of amygdala connectivity with Addenbrooke’s Cognitive Examination-Revised (ACE-R) in all participants. We investigated the relationship of z-scores and ACE-R scores within regions that showed significant connectivity differences between groups and correlation with the OSIT-J scores to show that some connectivity changes were also correlated with ACE-R scores. Regression analysis of amygdala connectivity with the ACE-R score in all participants masked by the contrast of the group comparison and OSIT-J result showed significance in the inferior parietal lobule, lingual gyrus, fusiform gyrus, and superior and middle temporal gyrus. All maps were corrected for multiple comparisons using FWEc p < 0.05 and CDT p = 0.001.
(TIF)

S6 Fig Program Listing. Source code of the Matlab script used to compute the correlation between a given seed ROI’s mean time series and that of the whole brain.
(DOCX)

S7 Fig MNI coordinates. List of areas (cluster peak’s MNI coordinate, cluster size, z-value, labels, and regional peak’s MNI coordinates) showing significant connectivity difference for all group-level comparisons and all seed ROIs.
(XLSX)
