# Supplementary figures and images for: Severe hyposmia and aberrant functional connectivity in cognitively normal Parkinson’s disease
Source: PLoS One. 2018 Jan 5;13(1):e0190072. doi: 10.1371/journal.pone.0190072 (PMC5755765; doi:10.1371/journal.pone.0190072)

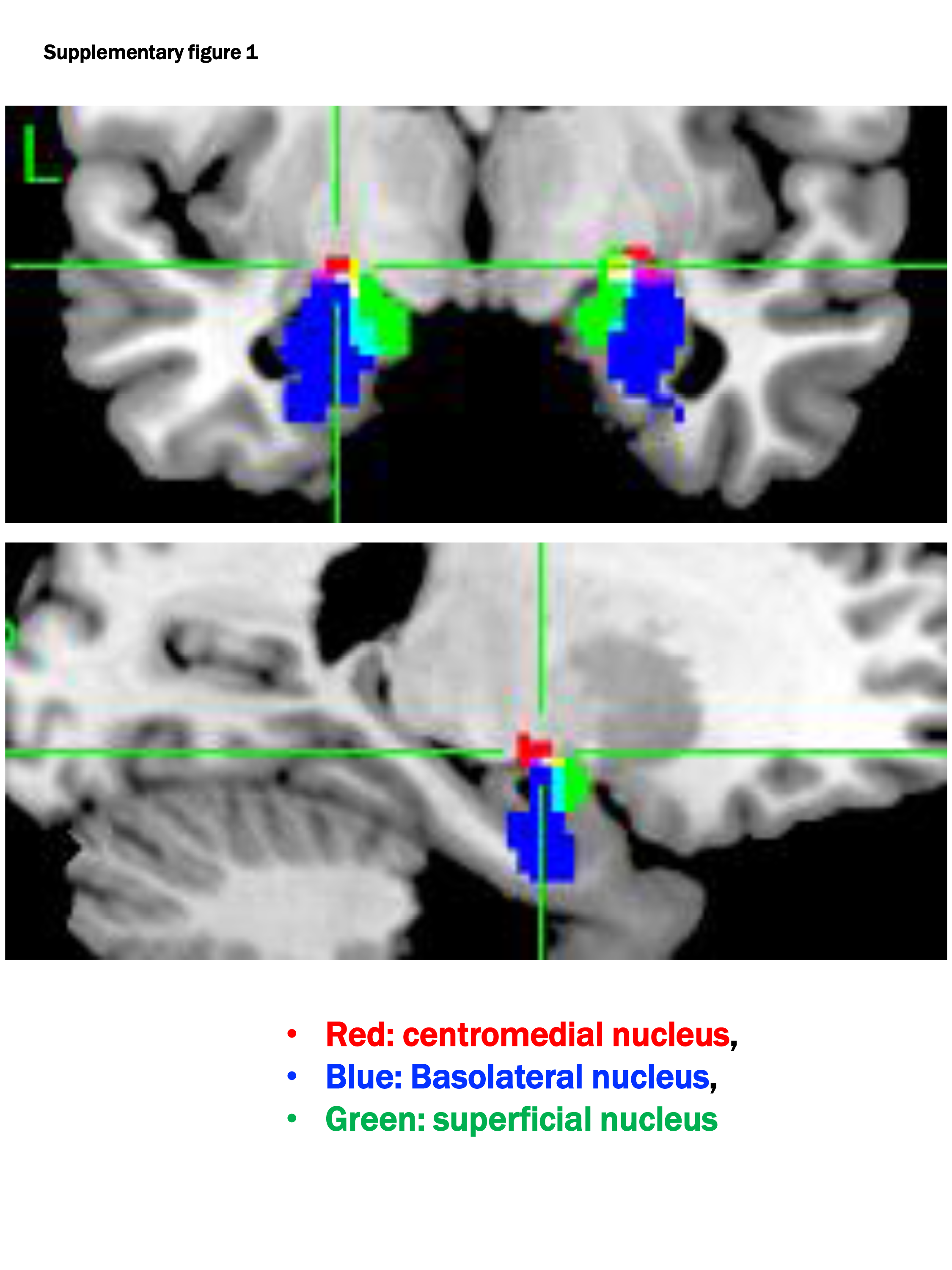

Supplement: S1 Fig — The amygdala was divided into six subregions: left and right centromedial amygdala, left and right laterobasal amygdala, and left and right superficial amygdala. These were used as seed regions. (TIF) [file pone.0190072.s001.tif]

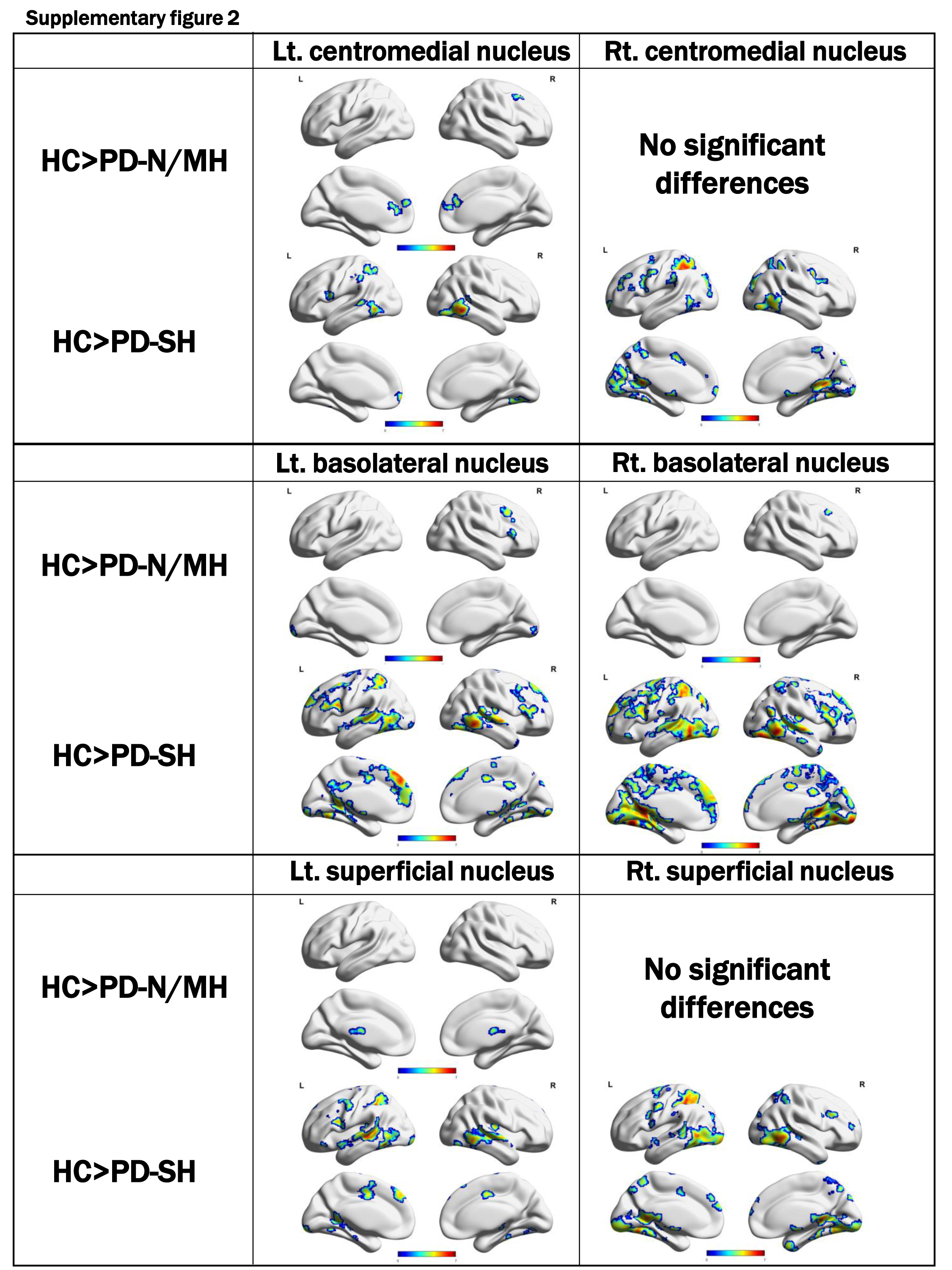

Supplement: S2 Fig — In the Parkinson’s disease with severe hyposmia (PD-SH) group, the amygdala nuclei showed widespread decreases in functional connectivity (FC) with other brain areas compared with healthy controls (HCs). The PD-N/MH group also showed decreased FC compared with HCs in three regions of interest (ROIs); however, the extent of these abnormal connectivity regions was limited compared with those of the PD-SH group versus HCs. All maps were corrected for multiple comparisons using FWEc p < 0.05 with CDT p = 0.001. (TIF) [file pone.0190072.s002.tif]

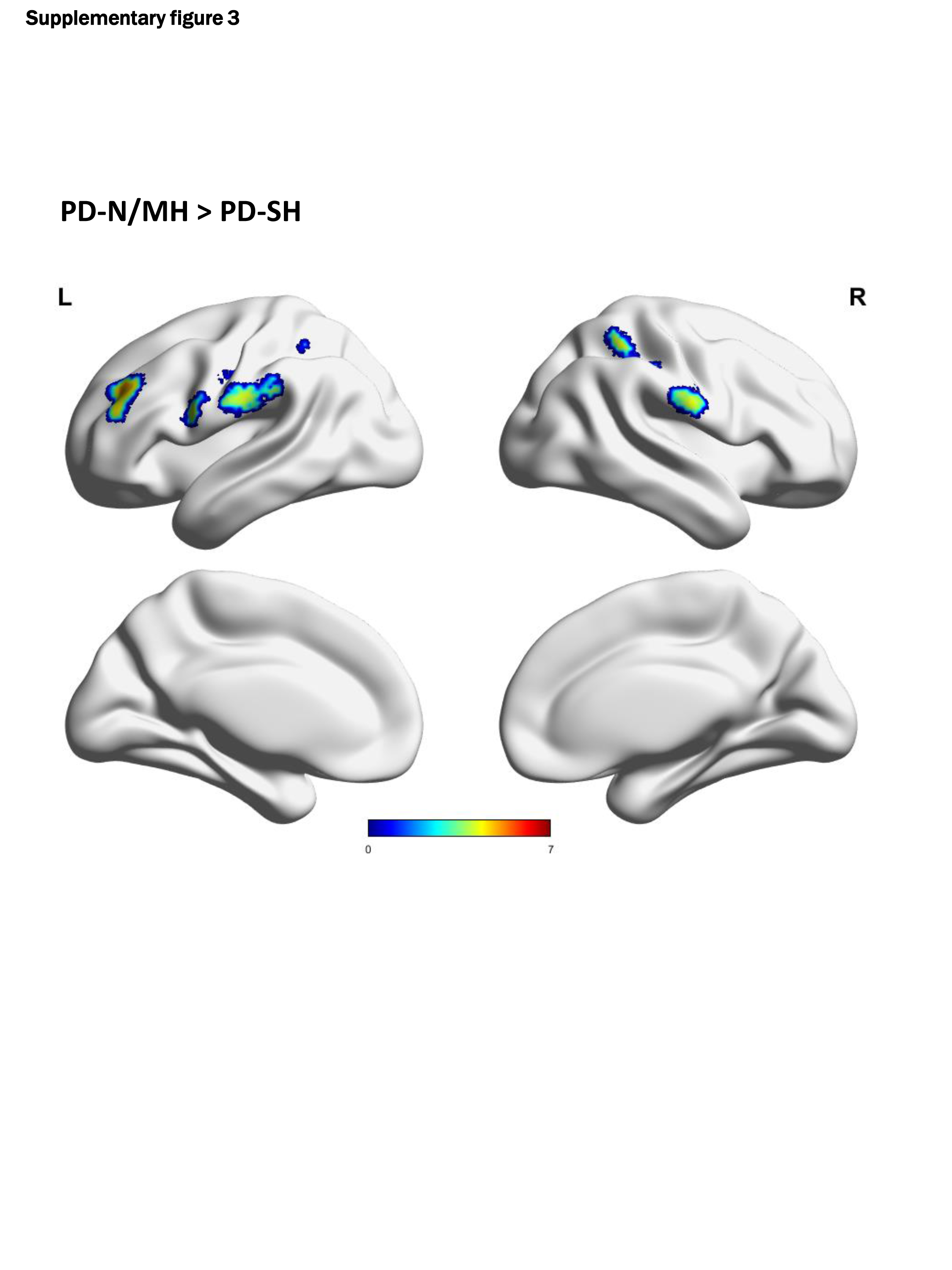

Supplement: S3 Fig — The PD-SH group showed decreased FC between the right centromedial and mainly dorsolateral prefrontal cortex and parietal cortex including left inferior frontal gyrus/BA9, postcentral gyrus, precentral gyrus, inferior parietal lobule/BA40, middle frontal gyrus/BA9, right inferior parietal lobule, postcentral gyrus/BA3, precentral gyrus, and paracentral lobule compared with the PD-N/MH group. However, there were no significant differences in FC of other amygdala seeds of interest. (TIF) [file pone.0190072.s003.tif]

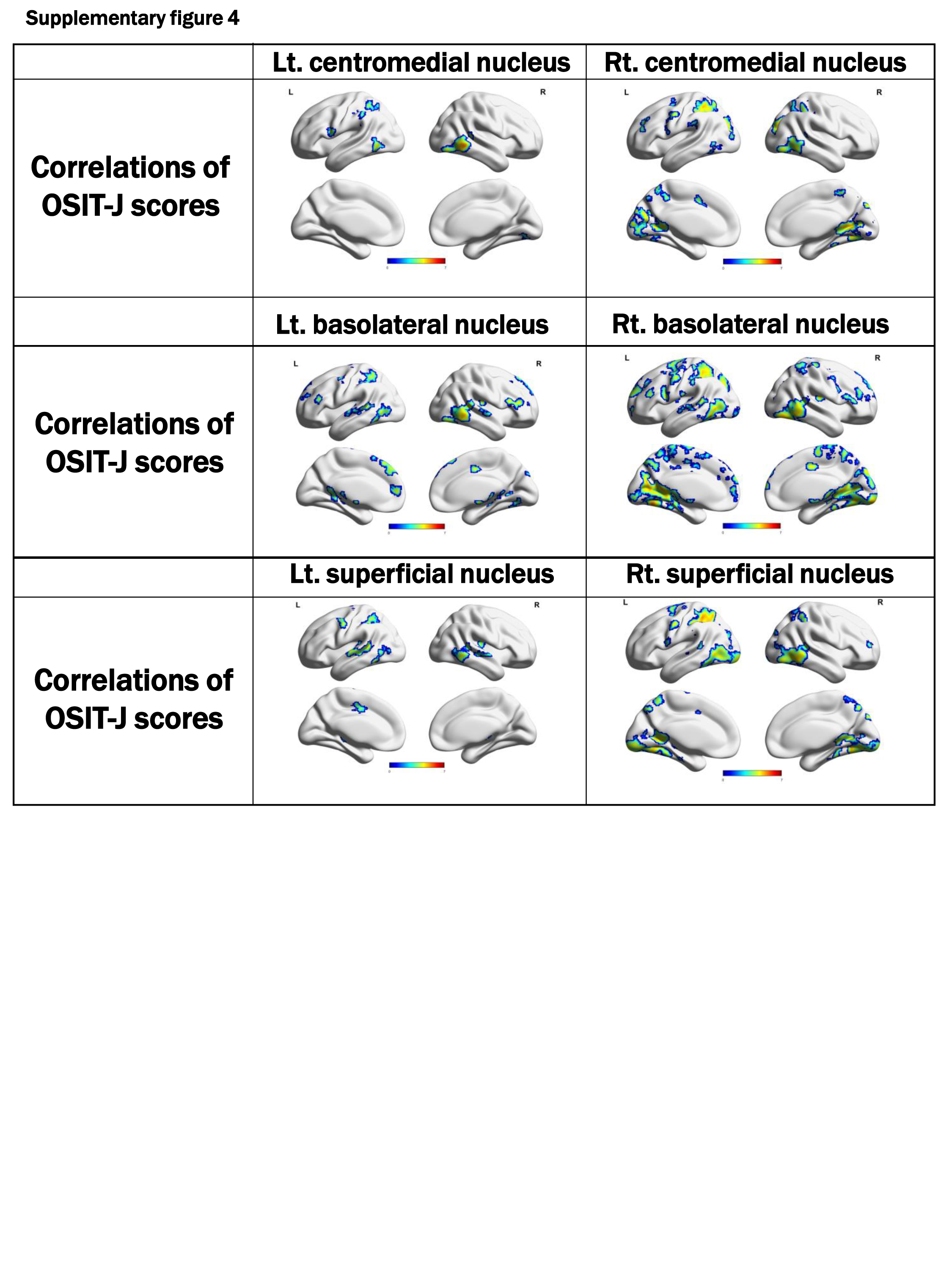

Supplement: S4 Fig — We investigated the relationship of z-scores and OSIT-J scores within regions that a showed significant connectivity difference between groups to demosntrate that some connectivity changes were also correlated with OSIT-J scores. We masked the result of the regression analyses using contrasts obtained from group comparisons (e.g., contrast map from HC > PD-SH). Most of the connectivity changes were correlated with the OSIT-J scores, signifying that these changes were related to hyposmia in all participants. All maps were corrected for multiple comparisons using cluster-level family-wise error (FWEc) p < 0.05 and cluster-defining threshold (CDT) p = 0.001. (TIF) [file pone.0190072.s004.tif]

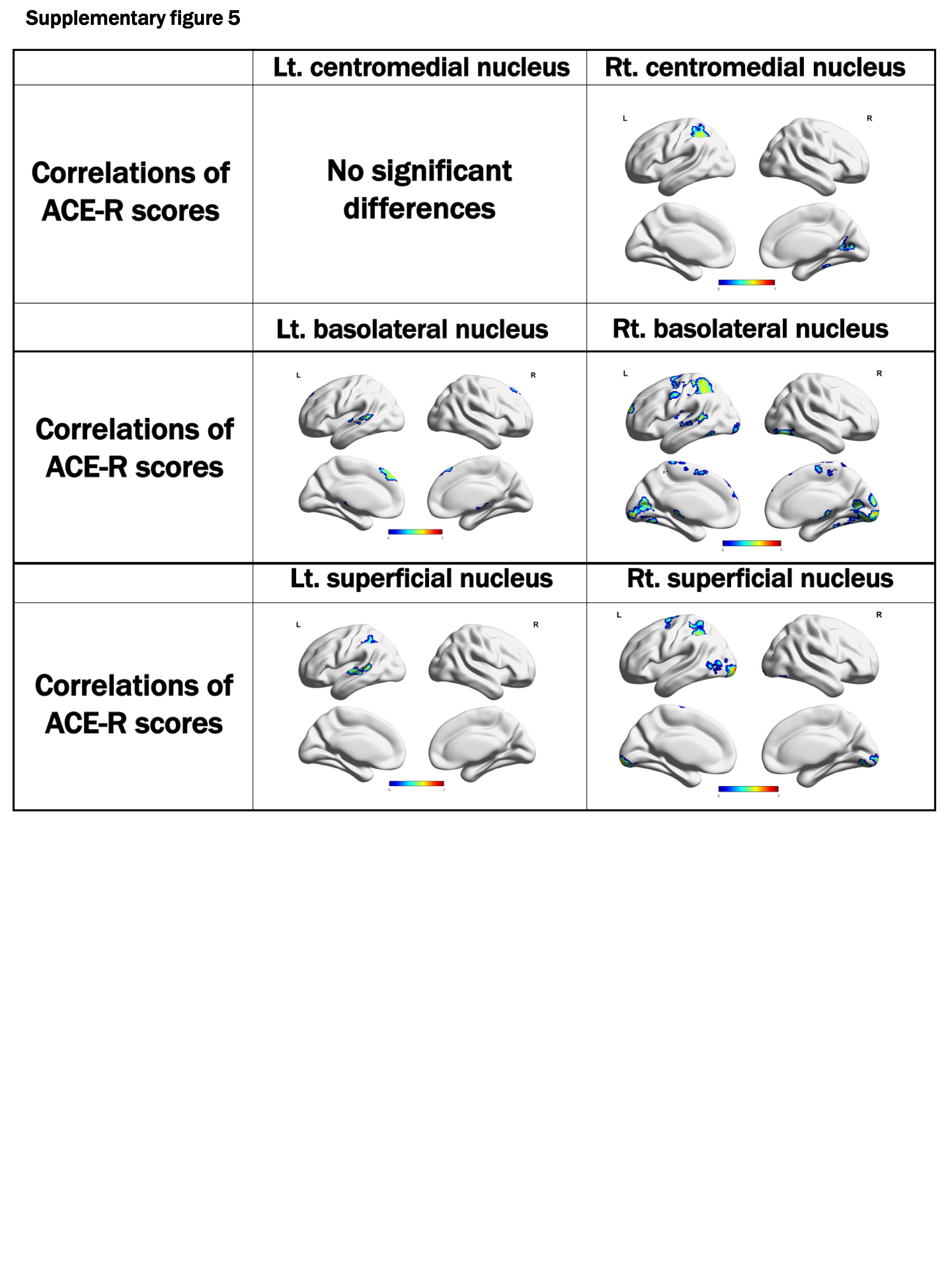

Supplement: S5 Fig — We investigated the relationship of z-scores and ACE-R scores within regions that showed significant connectivity differences between groups and correlation with the OSIT-J scores to show that some connectivity changes were also correlated with ACE-R scores. Regression analysis of amygdala connectivity with the ACE-R score in all participants masked by the contrast of the group comparison and OSIT-J result showed significance in the inferior parietal lobule, lingual gyrus, fusiform gyrus, and superior and middle temporal gyrus. All maps were corrected for multiple comparisons using FWEc p < 0.05 and CDT p = 0.001. (TIF) [file pone.0190072.s005.tif]
