# Supplementary material for: Severe hyposmia and aberrant functional connectivity in cognitively normal Parkinson’s disease
Source: PLoS One. 2018 Jan 5;13(1):e0190072. doi: 10.1371/journal.pone.0190072 (PMC5755765; doi:10.1371/journal.pone.0190072)
Supplement: S6 Fig — Source code of the Matlab script used to compute the correlation between a given seed ROI’s mean time series and that of the whole brain. (DOCX) [file pone.0190072.s006.docx]

Program List

function abk_funconn(infn,seedfn,opt)

% abk_funconn(infn,seedfn,opt)

%

% Function to compute correlation between seed ROI’s mean time series

% and that of the whole brain. Requires SPM and Matlab’s corr() function.

% infn = input filename (resting state fMRI data, NIFTI format)

% seedfn = filename of the seed ROI (NIFTI format)

% opt = options

% - maskfn = filename of the mask (NIFTI format)

% - offset = number of volumes to exclude (1:offset) (default: 0)

% - outpref = prefix for output filename

%

% coded by: E. Bagarinao (ebagarinao@met.nagoya-u.ac.jp)

% check number of input arguments, return if less than 2

if nargin < 3

help abk_funconn;

return;

end

offset = 0;

if isfield(opt,'offset')

offset = opt.offset;

end

% load the mask

if ~isfield(opt,'maskfn')

fprintf('abk_funconn: Missing required field ''maskfn'' in opt\n');

return;

end

volMask = spm_vol(opt.maskfn);

img = spm_read_vols(volMask);

idxMask = find(img(:) > 0.5);

clear img;

% read seed ROI image

volSeed = spm_vol(seedfn);

if ~isequal(volMask.mat,volSeed.mat)

fprintf('abk_funconn: Different image space in %s.\n',seedfn);

return;

end

img = spm_read_vols(volSeed);

idxSeed = img(:) > 0.5;

clear img;

% load the image

vol = spm_vol(infn);

% TODO: verify if the same header info as the mask

if ~isequal(volMask.mat,vol(offset+1).mat)

fprintf('abk_funconn: Different image space in %s.\n',infn);

return;

end

img = spm_read_vols(vol);

img(:,:,:,1:offset) = []; % remove offset images

sz = size(img);

% reshape image, row for time, columns for voxels

img = reshape(img,prod(sz(1:3)),sz(4))';

rseries = img(:,idxMask); % all time series within mask

refts = img(:,idxSeed); % reference time series within seedROI

mrefts = mean(refts,2); % mean of reference time series

% compute correlation

[rcff, pval] = corr(rseries,mrefts);

zcff = rtoz(rcff);

% save to file

[dn,fn1,~] = fileparts(infn);

fn1 = strrep(fn1,' ','_');

[~,fn2,~] = fileparts(seedfn);

fn2 = strrep(fn2,' ','_');

outpref = [fn2 '_' fn1];

if isfield(opt,'outpref')

outpref = opt.outpref;

end

% save mean time series

fname = fullfile(dn,['rts_' outpref '.txt']);

save(fname,'-ascii', 'mrefts');

% save correlation coefficient

fname = fullfile(dn,['rcff_' outpref '.nii']);

msk2nii(idxMask,opt.maskfn,fname,rcff,16);

% save z-value

fname = fullfile(dn,['zcff_' outpref '.nii']);

msk2nii(idxMask,opt.maskfn,fname,zcff,16);

% save pvalue

fname = fullfile(dn,['pval_' outpref '.nii']);

msk2nii(idxMask,opt.maskfn,fname,1-pval,16);

end

% compute z-transform

function zf = rtoz(img)

zval = 0.5*log((1+img) ./ (1-img));

ix = zval(:)==Inf;

zval(ix) = 0;

zf = zval;

end

% save data to nii

function msk2nii(idx,mskfn,outfn,data,dtype)

% idx is the index in the image where data will be assigned

% mskfn is the mask filename

% outfn is the output filename

% data is the data to store in the image in the indices identified by idx

% dtype is the datatype (default: 2 - uint8), check spm_type()

% check input arguments, assign default values

if nargin < 4

imdata = ones(1,length(idx));

dt = 2; % uint8

else

imdata = data;

dt = dtype;

end

% read header information

vol = spm_vol(mskfn);

% initialize image to write

img = zeros(vol.dim);

% assign data values to image identified by idx

img(idx) = imdata;

% prepare header information

tvol.fname = outfn;

tvol.dim = vol.dim;

tvol.mat = vol.mat;

tvol.pinfo = [1 0 352]';

tvol.dt = [dt 1];

tvol.descrip = 'generated: msk2nii.m';

% write image to file

spm_write_vol(tvol,img);

end
